# Supplementary material for: Real-Life Effectiveness of Mepolizumab on Forced Expiratory Flow between 25% and 75% of Forced Vital Capacity in Patients with Severe Eosinophilic Asthma
Source: Biomedicines. 2021 Oct 27;9(11):1550. doi: 10.3390/biomedicines9111550 (PMC8615088; doi:10.3390/biomedicines9111550)
Supplement: Supplementary file 1 [file biomedicines-09-01550-s001.zip › biomedicines-1417657-supplementary.pdf]

SUPPLEMENTARY MATERIAL

Table S1. Clinical, biological and functional effects reported during treatment with mepolizumab

	T0	T6	p-value (T0-T6)	T12	p-value (T6-T12)	T18	p-value (T12-T18)
**Clinical effects**							
Asthma control test	14.5(11;17.7) (n=97)	19(17;22.5) (n=94)	<0.0001	22(18.5;24) (n=70)	0.0002	23(20;24) (n=58)	0.5305
ACT >20, (%)	17 (17.5)	44 (46.8)	<0.0001	49 (70)	0.004	42 (72.4)	0.8457
Exacerbations (n=84)	4.2 ± 2.5	/	/	1.1 ± 1.6 (n=68)	/	/	/
OCS, (%)	54 (51.4)	19 (18.1)	<0.0001	10 (11.9)	0.3106	8(11.4)	>0.9999
**Biological effects**							
Blood eosinophils (cells/µL)	500 (350; 717)	80 (40;130)	<0.0001	70 (40; 110)	0.004	50 (30; 100)	0.0486
FeNO, ppb	41 (24; 61.5) n=73	42.5 (25; 78.7) n=68	ns	37 (23;53)	ns	33 (1.5;44)	ns
**Pulmonary function tests**							
FEV1, % of predicted	63.7 ± 17.9	71.9 ± 18.0	<0.0001	73.9 ±16.9	0.303	77.3±17.4	0.461
FVC, % of predicted	79.9 ± 16.2	85.3 ± 17.2	0.0001	86.6 ± 15.5	0.992	89.3 ±16.5	0.416
FEV1/FVC	67.5 ± 13.0	71 ± 13.4	<0.0001	71.9 ± 13.2	0.052	72.6±11.4	0.238
FEF25-75, % of predicted	32.7 ± 18.2	40.8 ± 21.3	<0.0001	45.3 ± 21.1	<0.0001	48.6±18.4	<0.0001

Data presented as Mean (SD), Median (IQR) and n (%).

Table S2. Effect of mepolizumab treatment in SEA patients according to blood eosinophilia.

	eos ≥400	eos <400	
	n= 67	n=38	p value
**Baseline**			
FEV1, % of predicted	60 (46-77)	68.5 (61.8-75.3)	**0.05**
FVC, % of predicted	80 (67-90)	83.5 (76.5- 92.3)	ns
FEV1/FVC	66 (56.7-71)	73 (67-83.3)	**0.0001**
FEF25-75, % of predicted	25 (14-45)	35 (23.5-48)	ns
Asthma control test	14 (11- 16)	15 (9- 20)	ns
**6 months**			
FEV1, % of predicted	73.5 (55- 87)	73 (64-81.5)	ns
FVC, % of predicted	88 (71.5- 96.5)	85 (79- 84)	ns
FEV1/FVC	69 (60.5-75.8)	75 (67.5-85)	**0.0035**
FEF25-75, % of predicted	35.5 (22.3-53)	37 (29.5-54.5)	ns
Asthma control test	18-5 (17-22)	20 (17- 23)	ns
**12 months**			
FEV1, % of predicted	71.5 (56.3- 89.3)	73(66-85)	ns
FVC, % of predicted	86 (74-100.8)	87 (81- 92)	ns
FEV1/FVC	69 (63.1-75)	76 (72- 85)	**0.001**
FEF25-75, % of predicted	45 (24-58)	47 (30-58)	ns
Asthma control test	22 (19-24)	22.5 (15.3-24)	
**18 months**			
FEV1, % of predicted	75 (64.3-87.8)	76 (71-91)	ns
FVC, % of predicted	90.5 (76- 98)	88 (80- 97)	ns
FEV1/FVC	69 (63-75)	78 (72-82)	**0.001**
FEF25-75, % of predicted	47 (27-61)	58 (32-66)	ns
Asthma control test	23 (21-24)	22 (15.3- 24)	ns
**exacerbation rate**			
Baseline	4 (3- 5)	3 (2 - 5)	ns
12 months of mepolizumab	0 (0- 2)	0 (0-1)	ns
**Difference from baseline**			
**After 6 months**			
ΔFEV1, % of predicted	9.5 (1-18.3)	6 (1-10)	ns
ΔFVC, % of predicted	5 (-4- 15.3)	3 (-0.5 - 8)	ns
ΔFEV1/FVC	4.1 (1.7-8.2)	2 (-0.5 - 5)	ns
ΔFEF25-75, % of predicted	5 (1-17)	6 (0-13.5)	ns
ΔAsthma control test	4 (2.8- 7.3)	4 (2- 6.5)	ns
**After 12 months**			
ΔFEV1, % of predicted	12 (6.3-19)	7 (0-12)	**0.0148**
ΔFVC, % of predicted	5.5 (-1, 16.8)	2 (-1 - 8)	ns
ΔFEV1/FVC	5.4 (1-11.4)	3 (1- 6)	ns

∆FEF25-75, % of predicted	13 (5-22.3)	8 (3-12)	**0.0437**
∆Asthma control test	8 (5- 9)	5 (1- 8)	**0.0445**
**After 18 months**			
∆FEV1, % of predicted	11 (4.3- 19.5)	9 (4-14)	ns
∆FVC, % of predicted	5 (-1, 14.5)	7 (1- 11)	ns
∆FEV1/FVC	5.1 (1.8-12.3)	5 (1- 8)	ns
∆FEF25-75, % of predicted	12 (5.3-22.5)	15 (9-20)	ns
∆Asthma control test	8 (4-10)	5 (2-7)	**0.0336**

Table S3. Effect of mepolizumab treatment in SEA patients according to OCS use.

	**OCS**	**No OCS**	
	n= 54	n=51	p value
**Baseline**			
FEV1, % of predicted	62 (46.7- 73)	67 (54-83)	ns
FVC, % of predicted	79.5 (66.3- 86.3)	86 (69-95)	**0.0264**
FEV1/FVC	65.5 (55-74.5)	70 (65-80.5)	**0.0094**
FEF25-75, % of predicted	26 (13.5- 43.5)	32 (23-48)	**0.0547**
Peripheral blood eosinophils, cells/µL	560 (365 - 820)	400 (330-600)	**0.0114**
Asthma control test	14 (11- 16)	14 (9- 20)	ns
**6 months**			
FEV1, % of predicted	71.5 (54.7-82.7)	79 (59.5-86.5)	ns
FVC, % of predicted	85.5 (73.5-93)	88(76-98.5)	ns
FEV1/FVC	69 (59.3-76)	73 (66-84)	**0.0097**
FEF25-75, % of predicted	35.5 (21.7- 53.3)	37 (30- 55)	ns
Asthma control test	18.5 (17- 21)	20 (18- 23)	ns
**12 months**			
FEV1, % of predicted	71(60-84)	74 (62-92.5)	ns
FVC, % of predicted	85 (75-94)	89 (74.8-102)	ns
FEV1/FVC	69 (55-73)	75 (68.2-85)	**0.0005**
FEF25-75, % of predicted	48 (22-58)	45 (30- 59)	ns
Peripheral blood eosinophils, cells/µL	80 (47.5- 122.3)	60 (30- 105)	ns
Asthma control test	21.5 (18.8- 24)	22 (18- 24)	ns
**18 months**			
FEV1, % of predicted	75 (54.7-82.7)	77 (65.5- 92.5)	ns
FVC, % of predicted	87.5 (75.7-93)	91 (77.5- 103)	ns
FEV1/FVC	71.1 (61.7-75.5)	76 (68.2-79.7)	**0.0236**
FEF25-75, % of predicted	52 (39.8- 62.3)	47.5 (31.3- 62.5)	ns
Asthma control test	22 (20- 24)	23 (19- 24)	ns
**exacerbation rate**			

Baseline	4 (3-5)	3 (2- 5.3)	ns
12 months of mepolizumab	1 (0- 2)	0 (0- 1)	ns
**Difference from baseline**			
**After 6 months**			
ΔFEV1, % of predicted	6.5 (0.7-16)	6 (2-17.5)	ns
ΔFVC, % of predicted	3.5 (-1.5 -32.3)	3 (-3.5- 13.5)	ns
ΔFEV1/FVC	4 (-0.4 -8)	4 (0- 8)	ns
ΔFEF25-75, % of predicted	6 (-0.5 - 17.5)	5 (2.5 -9)	ns
ΔAsthma control test	4 (3- 6)	4 (0- 9)	ns
**After 12 months**			
ΔFEV1, % of predicted	10 (2-15)	9.5 (3.3- 18.5)	ns
ΔFVC, % of predicted	3 (-2 - 13)	5 (0-13)	ns
ΔFEV1/FVC	5 (0-7)	4 (1- 11)	ns
ΔFEF25-75, % of predicted	14 (4.8- 23)	7 (5- 13)	**0.0418**
ΔAsthma control test	6 (4.8- 8)	2 (6.5- 11)	ns
**After 18 months**			
ΔFEV1, % of predicted	10.5 (4.7-15)	8 (2.5-17.5)	ns
ΔFVC, % of predicted	4 (-0.3 - 12)	7 (-0.5 - 16)	ns
ΔFEV1/FVC	5.1 (0.75- 11)	5 (1-12)	ns
ΔFEF25-75, % of predicted	18 (9- 34)	11 (6- 18.5)	**0.0445**
ΔAsthma control test	7.5 (5- 9)	5 (2- 10.5)	ns

Figure S1 – Comparison among subgroups of patients with different baseline eosinophil levels.

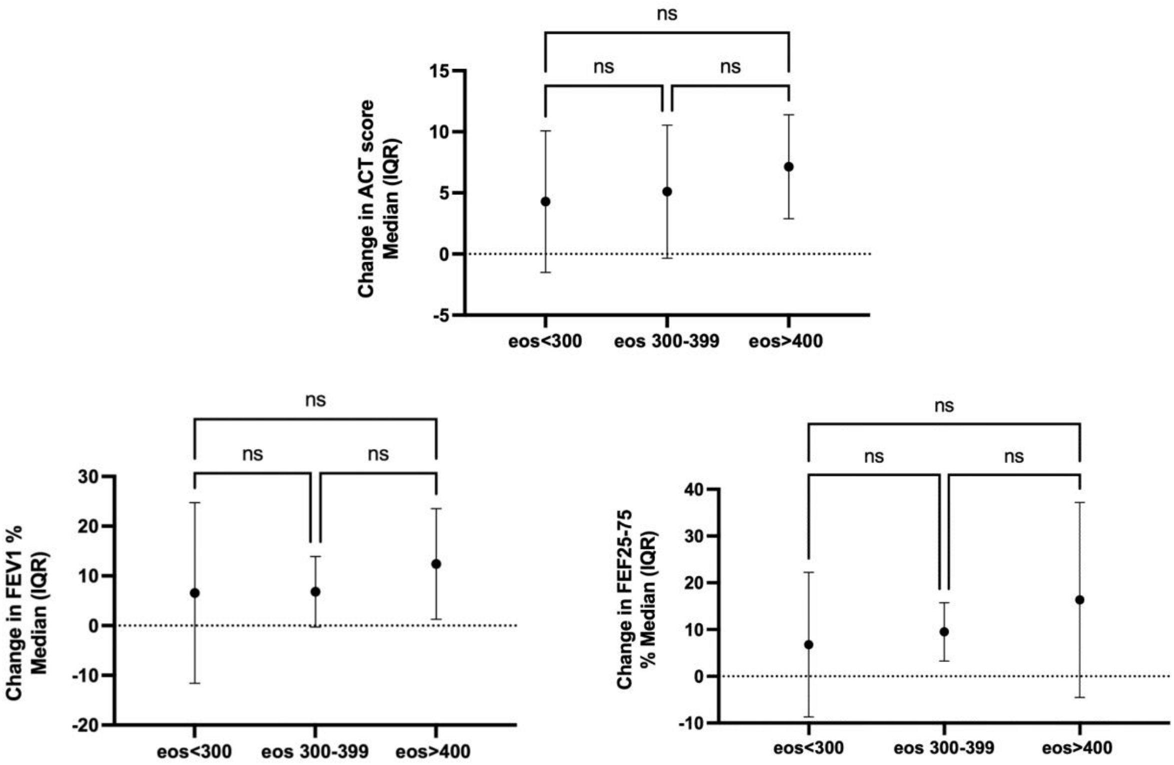

Figure S1. Effects of mepolizumab, according to level of blood eosinophilia (blood eosinophils <300 cells/μL, between 300-399 cells/μL and ≥400 cells/μL) in regard to a) change in ACT score, b) change in FEV1, c) change in FEF25-75 at 12 months follow-up compared to baseline.
